# Process Evaluation of Maternal, Child Health and Nutrition Improvement Project (MCHNP) in the Eastern Region of Ghana: A Case Study of Selected Districts

**DOI:** 10.1155/2020/1259323

**Published:** 2020-09-18

**Authors:** Solomon Boamah Amponsah, Eric Osei, Moses Aikins

**Affiliations:** ^1^Research and Development Unit, Regional Health Directorate, Eastern Region, Ghana; ^2^Department of Population and Behavioural Sciences, School of Public Health, University of Health and Allied Sciences, Volta Region, Ghana; ^3^Department of Public Health Graduate School, Yonsei University, Seoul, Republic of Korea; ^4^Department of Health Policy, Planning and Management, School of Public Health, College of Health Sciences, University of Ghana, Ghana

## Abstract

**Background:**

Maternal, Child Health and Nutrition improvement Project is a World Bank-funded project implemented in all then ten regions of Ghana, which aims at improving access and utilization of community-based maternal, child health, and nutrition services in order to accelerate progress. This study is aimed at determining the implementation status of the project in the Eastern region by evaluating the processes involved and identifying implementation barriers from the perspective of implementors.

**Methods:**

The study was a cross-sectional in design and employed a quantitative data collection approach in ten Community-based Health Planning and Services (CHPS) centres in five districts in the region. The project coordinators and Community Health Officers were interviewed using a structured questionnaire. The project implementation reports at the facility level were reviewed using a checklist. Tertile statistic was used to describe the status of the project implementation.

**Result:**

The finding from this study indicated “complete implementation status” for maternal, child health, and nutrition activities of the project. However, none of the facilities evaluated had satisfactorily implemented all the governance processes and were therefore rated as “partially complete.” The main implementation barriers emerged from the study were related to restrictions placed on the use of project funds and delays in the fund disbursement to CHPS facilities.

**Conclusion:**

The evidence gathered from the study showed very good implementation status for community-led maternal and child health service delivery, indicative of a positive response to the guidelines by service providers at the periphery and can have positive impact on the project's objectives and goals. Governance component of the project, however, revealed inadequate alignment with guidelines which might have been influenced by the lack of knowledge as a result of lack of training for implementers. This therefore calls for in-service training and improved supportive supervision at both administrative and service delivery levels.

## 1. Background

Ghana has made a significant progress over the years in improving health outcomes, especially reducing maternal and child mortalities [1]. There has been a decline of infant mortality rate by 47% since 1988, from 77 deaths to 41 per 1,000 live births in 2014. An even more remarkable decline was noticed in under 5 mortality, which fell by 61% from 155 deaths to 60 deaths per 1,000 live births over the same period [2]. Total fertility rate (TFR) also declined from 6.4 children per woman in 1988 to 4.2 children per woman in 2014 [3]. Statistics from 2014 Demographic and Health Survey (DHS) also indicated increased antenatal and postnatal care, improved delivery practices, and improved maternal health [2]. The considerable progress made by Ghana in reducing maternal mortality has largely been supported by several initiatives by the United Nations (UN) and other partners. Such initiatives included framework on the Millennium Development Goals (MDGs), safe motherhood, vitamin A supplementation trials, and others [1].

The Ministry of Health, in 2011, acknowledged that though Ghana has made some progress in the past, barriers of inequalities, geographical disparities, and sustaining the progress remain unaddressed [4]. As a result, Ghana is not on track to meet all health-related Sustainable Development Goals (SDGs) targets. For the country to accelerate progress on the reduction of maternal and child mortalities and improve health outcomes, a well-targeted approach which expands access to cost-effective interventions, focusing on women and children from poor homes and rural areas must be adopted [5]. Recognising this, the World Bank funded the Maternal, Child Health and Nutrition improvement project (MCHNP), which is been implemented in all then ten regions of the country since 2015 to accomplish intended enhancements in health and nutrition outcomes to further reduce maternal and child mortalities.

The project, which is expected to end in 2020, builds on the Community-based Health Planning and Services (CHPS) initiative established by Ghana in an effort to address disparities in access to maternal and child health services and strives to offer funding to reinforce key interventions, eliminate blockades to access health, and strengthen responsibility and institutional ability. There are two mutually reinforcing components of the project, namely, service delivery and capacity building. The service delivery component centres on strengthening supply, creating demand, and increasing ownership and accountability of district-level stakeholders, outreach workers, community leaders, and household members. The component supports the uptake of a package of essential community nutrition and health actions (ECNHA) and addresses gaps in knowledge and community practices such as reproductive behaviour, nutritional support for pregnant women and young children, recognition of illness, home management of sick children, disease prevention, and care-seeking behaviour. The capacity building component provides a rolling programme of training and orientation, spread over the lifetime of the project. Community Health Officers (CHOs) and Community Health Volunteers (CHVs) are oriented and trained on specific health services and the contents of the CHOs' Training Manual [5].

In Ghana, District Health Management Teams (DHMTs) are required to enhance the skills of Community Health Nurses (CHNs) (or other cadre of staff) to prepare them to provide preventive and curative care while residing in the community. These health staff, known as CHOs, ride on motorcycle to travel from compound to compound to provide door-to-door health services to individuals and households and are expected to cover a catchment area of about 3,000 residents [6]. CHOs' activities are supported by CHVs lay individuals with varied background who are selected by the community to represent them. CHVs receive brief training and work with the CHOs in the communities to assist with community mobilization, the maintenance of community registers, and other essential activities, with the potential to supplement the formal health system in the effort to achieve Universal Health Coverage in low- and middle-income countries (LMICs) [7].

Processes ensuring that MCHNP achieves the intended results are equally important as the results themselves. In the absence of a methodical evaluation, implementers are likely not to know whether the project scheme is being successful. An evaluation of MCHNP during implementation will serve as a tool to advocate for ways to improve or expand processes and point out strengths and flaws. There is also the need to identify barriers in the execution of the project in the region. In this context, a process evaluation was undertaken in the Eastern region of Ghana to provide information on the implementation status of the project and possible barriers affecting its implementation in the region.

## 2. Materials and Methods

### 2.1. Study Design and Setting

The study was a cross-sectional descriptive study, which employed quantitative approach to determine the current status of the key processes (governance, maternal health, child health, and nutrition services) of the MCHNP in the Eastern region of Ghana. The region is the sixth largest region in Ghana with a land area of 19,323 kilometres square. It has a population of 3,171,743, made up of 49% males and 51% females. There are 26 districts in the region, which are further demarcated into 183 administrative subdistricts; 18 of the 26 districts have at least one hospital. Other levels of health facilities abound in all the districts in the region. MCHNP is being implemented in all 183 subdistricts and 828 CHPS centres in the region. The project covers the entire population of the region.

### 2.2. The Project Design and Implementation

The project was designed to address the inequity gap in order to increase utilization of maternal and child health services. Within the participating communities, the project targets pregnant women and children under 2 years of age. Besides, the project also benefits other people in the community, especially children under 5 years, with wide range of community-based interventions such as the promotion of family planning, early registration of pregnant women for antenatal care, skilled delivery, exclusive breastfeeding, birth registration, and growth promotion among others [5].

### 2.3. Project Management

The Regional Director of Health Services (RDHS) is accountable for implementing and tracking the project operations at the regional level, supported by the Director of Public Health, the Nutrition Officer, and regional Disease Control Officer. In line with the operating guidelines drawn up by Ghana Health Service (GHS), the District Director of Health Services (DDHS) coordinates the development and implementation of the district action plan for subprojects as well as monitoring project indicators, supported by the DHMT. For districts to develop context-relevant implementing strategies, the guidelines provide enough flexibility.

The main change agents in the project are the CHO's and CHVs, who carry out outreach programmes, home visits, and promote development operations. This initiative promotes the current community structures to mobilize members of the society, promote the selection and monitoring of community volunteers, and promote the monthly operations through periodic leadership conferences, which discuss advancement in the community. The volunteers help organize periodic community meetings to review the implementation process of the project. The project also utilizes current local structures to get community leaders to take responsibility for health and dietary problems in the community.

### 2.4. Sampling and Study Population

The study purposefully selected 3 administrative levels—one Regional Health Directorate, five District Health Directorates, and ten (10) functional CHPS centres. Thus, a total of 16 facilities were evaluated. A simple random sampling method was used to select 5 districts, and in each district, 2 CHPS centres involved in the project were sampled randomly. At the regional and district levels, the project coordinators were interviewed. At the CHPS level, the senior-most CHO who had been at the centres since the beginning of the project at the facility was interviewed. In a situation where there was more than one senior CHO, the one in-charge of the centres was interviewed.

### 2.5. Data Collection

Data was collected in the fourth year of the project implementation by trained research assistants. A structured questionnaire was used to collect the data on the demographic characteristics of the facilities, MCHNP activities being implemented, and the views of participants on barriers to MCHNP implementation. All interviews were done in English language and lasted for about 30 to 40 minutes. Prevailing processes as reviewed in the records of MCHNP implementation at the facility level were compared to the original project processes outlined in the project's implementation guideline. Open-ended questions were used to elicit information from respondents on the barriers to the project implementation from the implementer's perspective.

### 2.6. Measurements and Data Analysis

**Figure 1:**
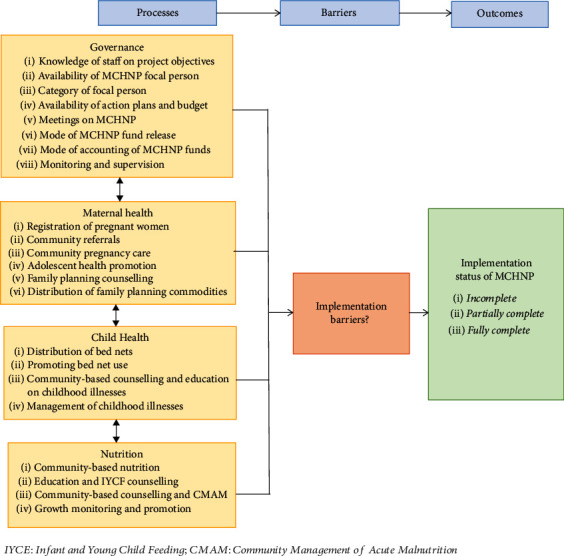
Conceptual framework showing the relationship between MCHNP processes, barriers, and outcomes. IYCE: infant and young child feeding; CMAM: community management of acute malnutrition.

The data was entered into a web-based data collection application (ODK_ona.io) and were checked for consistency and accuracy. MCHNP governance processes were determined by evidence of implementing MCHNP governance activities as outlined in the implementation guideline, an official appraisal document of the International Development Association Project. Maternal, child health, and nutrition processes were also determined by evidence of implementing MCHNP activities as outlined in the guideline. There are 8, 6, and 4 key activities expected to be implemented under Maternal, Child Health and Nutrition components, respectively.  Figure 1 shows the processes that define the outcome of the MCHNP implementation status and key implementable activities according to the guideline. Tertile descriptive statistic was used to determine the status of MCHNP implementation for all components studied. The measure had three percentile cutoff points; a percentage score of <37.5% indicated “incomplete process”; scores of 37.6-75% indicated “partially completed process,” and a score of 75.1-100% indicated “fully completed process.” To obtain the score for each key activity, the number of prevailing activities for each component under implementation was divided by the total number of activities in the MCHNP implementation guidelines and then multiplied by 100 to arrive at a percentage score. Analysis was carried out using the Microsoft Excel. Barriers to the implementation of MCHNP emerged from the interviews as enumerated by respondents. This was analysed by grouping same and very similar responses into major themes that represented the barriers and presented in a word cloud.

### 2.7. Research and Evaluation Framework

A conceptual framework (Figure 1) was used to illustrate the relationship between the project's processes, barriers, and implementation outcomes. Barriers at any stage of the implementation process for any of the activities may influence the implementation status, causing them to be either partially complete or incomplete. Lack of or inadequate knowledge on the project guidelines by implementers, for example, may result in nonadherence to guidelines, which may lead to some of the activities prescribed in the guidelines partially implemented or not implemented at all. Additionally, inadequate funding and lack of transport may impact negatively on supportive supervision to the peripheries. Barriers such as inadequate health personnel, lack of supplies, lack of community participation and commitment, and delays in release of funds may influence the implementation status.

### 2.8. Ethical Issues

The study obtained approval from the Ethics Review Committee of the Research and Development Division, Ghana Health Service (ref: GHS-ERC-035/06/19). Written permissions were obtained from the Eastern Regional Health Director and the Directors of the participating districts to conduct the evaluation and publish results. Additionally, written informed consent was sought from all respondents before the data were collected.

## 3. Results

Table 1 presents the characteristic of the facilities evaluated. Ten (10) CHPS centres, serving a total population of 36,581 from five (5) districts, were evaluated on the implementation of the MCHNP project. All but one (Nyamekrom opened in 2016) CHPS centre had been in operation for more than 5 years hence had been in operation since the inception of the project in 2015. Two Community Health Officers (CHOs) on the average worked in each facility, and the mean monthly out-patient (OPD) attendance was 136 patients, with Suhum Urban seeing the highest number of out-patients (283) and Chinto CHPS having the lowest (50).

### 3.1. Implementation Status

**Figure 2:**
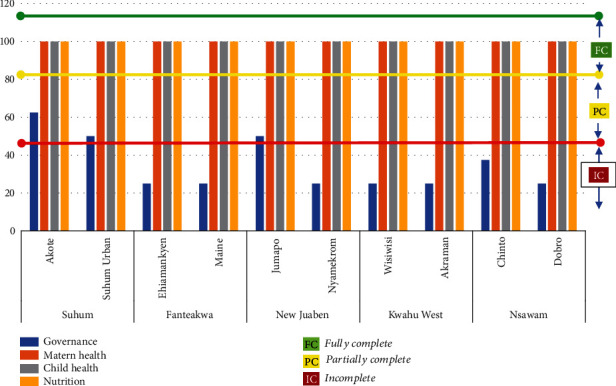
Status of MCHNP implementation processes.

Figure 2 presents the implementation status of all components and activities of the project. Akote CHPS centres had the highest score (62.5%) for governance, followed by Suhum Urban and Jumapo CHPS (50%) and then Chinto (37.5%) (Table 2), indicating partially complete implementation status. The rest of the CHPS centres (6; 60%) scored 25%, which indicated incomplete implementation status. The overall average score for MCHNP governance process was 43.8%. Based on this score, the status of governance processes was classified as partially complete. The implementation of MCHNP processes showed a fully completed implementation status for maternal, child health, and nutrition processes, with a score of 100% each for all facilities evaluated.

### 3.2. MCHNP Governance Processes Scores by CHPS Centres

Table 2 shows evidence of implementing MCHNP governance activities at the facility level as outlined in the implementation guideline. All districts evaluated had focal persons to coordinate MCHNP activities in all CHPS facilities in the district; however, none of these focal persons by label were observed to be of the prescribed cadre as required by the MCHNP guideline. No CHPS centres at the time of visit had documented evidence of plans and budget specific to the MCHNP project. Only one (10%) of the focal persons was able to mention all the MCHNP project objectives. Less than half (30%) (Akote, Jumapo, and Chinto) had documented evidence of quarterly MCHNP meetings conducted; Akote and Suhum Urban CHPS (20%) received funds through the prescribed mode as in the guideline, and 2 (20%) (Suhum Urban and Jumapo CHPS) received supportive supervision from the higher level within the quarterly timelines as required by the project. In all, three (3) of the facilities scored 50% and above of the governance processes, the rest scored 37% and below. The overall average score for governance process was 43.8%. Based on this score, the status of governance processes was rated as partially complete.

### 3.3. MCHNP Maternal Health Processes Scores by CHPS Centres

According to the evidence of implementing MCHNP maternal health activities as outlined in the MCHNP implementation guidelines, all the ten (10) facilities scored 100%. This indicates the overall status of MCHNP maternal health processes as fully completed as shown in Table 3.

### 3.4. MCHNP Child Health Processes Scores by CHPS Centres

As show in Table 4, all the facilities showed records on implementing MCHNP child health activities as outlined in the MCHNP implementation guidelines. The 100% score therefore established the status of MCHNP child health processes as fully completed in the facilities.

### 3.5. MCHNP Nutrition Processes Scores by CHPS Centres

According to the evidence of implementing MCHNP nutrition activities as outlined in the MCHNP implementation guidelines, all the 10 facilities scored 100% (Table 5). The overall score indicates that the status of MCHNP nutrition process was fully completed.

### 3.6. Barriers to MCHNP Implementation

**Figure 3:**
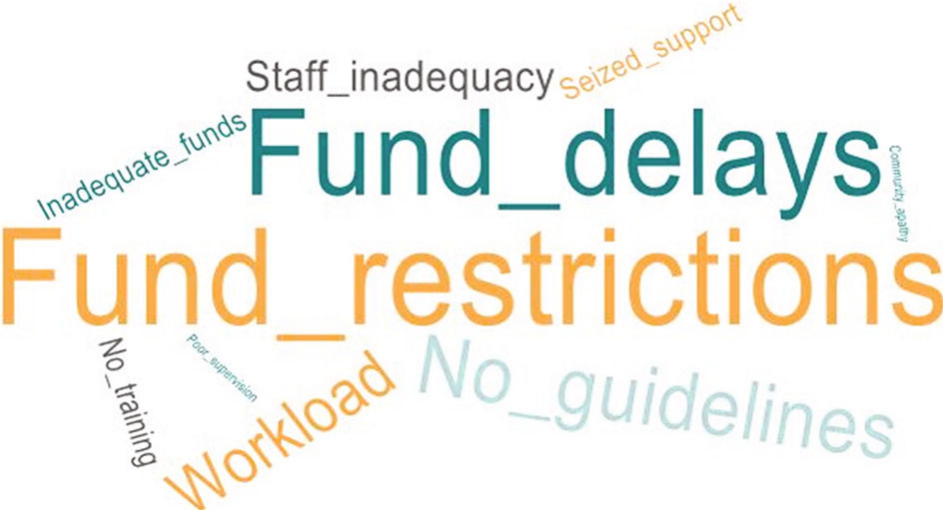
Word cloud showing barriers of MCHNP implementation.

All facilities assessed mentioned the delay in the release of funds and restrictions placed on the use of the MCHNP funds as barriers to implementation. The facilities did not have the option to use the MCHNP funds for any other purpose aside what it has been prescribed or budgeted for, example, facilities could not use the MCHNP funds to purchase equipment such as the BP apparatus or thermometers needed in basic service delivery. Additionally, 68.8% of the centres did not have MCHNP implementation guidelines, and 62.5% mentioned inadequate technical staff to carry out planned activities. Both the regional and district coordinators said there was no structured training or orientation on the project for implementers and coordinators before it was rolled out. The rest of the barriers identified were inadequate funding, poor community participation, and apathy in supporting activities by the District Health Directorates when MCHNP funds were depleted (Figure 3).

It was observed in most of the facilities (70%) that the lower the score on MCHNP governance, the higher the number of barriers mentioned. This was more pronounced in six (6) of the facilities. Furthermore, the Pearson correlation test found a significant correlation between MCHNP governance scores and the number of barriers identified (*r* = −1.00). The relationship showed that where there was a decrease in governance scores, the number of barriers increased. Similarly, it was noticed in most of the District Health Directorates (60%) that the lower the score on MCHNP governance, the higher the number of barriers mentioned. However, the Pearson correlation test found a less significant correlation between MCHNP governance scores and the number of barriers identified by MCHNP coordinators at the district level (*r* = 0.01).

## 4. Discussion

This study examined the implementation status of Maternal, Child Health and Nutrition improvement project, funded by the World Bank in Ghana. The interventions were evaluated in the context of the project's first components, namely, community-based maternal and child health and nutrition interventions. We assessed the implementation status of governance, maternal health, child health, and nutrition interventions and were classified as fully complete, partially complete, or incomplete implementation using a scoring system.

First, the status of implementation of governance activities such as plans and budget, qualified project focal persons, and meetings with stakeholders were found to be partially complete. None of the facilities evaluated had fully implemented all governance processes. It was observed that all districts, by extension CHPS centres, had focal persons for the MCHNP project; however, none of these focal persons by label were observed to be of the prescribed cadre as required by the MCHNP guideline. All the districts evaluate had either one of the following staff—Disease Control, Public Health, and Nutrition Officers coordinating the project both at the district and CHPS centres levels. In contrast to the MCHNP guideline, the District Director was required to act as the focal person in the district for the project and assist in the execution and supervision of the project's activities [5]. Considering the overwhelming roles of the District Directors of Health Services, however, it was appropriate and normal practice for the Directors to designate some of their roles to these technical officers to play and report back to them.

However, the finding from this study raises a question of whether the designated focal persons received the right orientation or training to be able to perform as expected. This is because it was observed that only one of the MCHNP focal persons was able to correctly enumerate all the MCHNP project objectives. This could be due to the absence or inadequate in-service training and orientation for the focal persons on their new roles and responsibilities as related to the project, as mentioned by some of the coordinators.

As part of the MCHNP institutional and implementation arrangements, Health Service Director in the district (DDHS) shall, following the operational rules prepared by the GHS headquarters, coordinate the preparation and executory district action plan for subprojects. The guidelines provide districts with ample flexibility in developing contextual implementing strategies [5]. However, the finding of this study showed that though the districts and facilities had general district or facility action plans for the year, a documented strategic plans and budget specifically for the MCHNP were not sighted. This finding raises concerns about the bases for which funds were released and how much was released to a CHPS centre without a guiding plan and budget. The absence of the action plans could also result in some of the project objectives not being met.

As part of the MCHNP administrative mechanisms, the project's main agents for change are Community Health Officers (CHOs) and Community Health Volunteers (CHVs). The volunteers are to support and to organise quarterly meetings with community stakeholders to discuss the implementation of the project interventions and to monitor its progress. The project uses local structures to promote chief and elder gatherings to establish a platform for ownership and accountability to address problems influencing the health and nutritional status of the community. In this context, we assessed evidence of meetings, and we found that only 3 of the CHPS centres had evidence of at least one meeting held on MCHNP in the first quarter of 2019. This suggests that meetings with the community and CHOs to review the progress of the project operations are nearly nonexistent in some communities and are likely to have an impact on the sustainability of the project.

Under component 1.2 of the community performance-based financing of MCHNP [5], the Community-based Performance Financing Program shall be assigned funding in the context of the project. CHPS Centre teams shall open bank accounts with rural banks located close to them for monies to be transferred from Ghana Health Services (GHS) to those accounts for the project implementation. The lead CHO and the lead volunteer in the teams will be the joint signatories for receipt of funds. On the contrary, this study found that apart from 2 facilities that received monies via their bank accounts, all the other 8 CHPS centres received cash directly from their Directorates or Subdistricts which in most instance delays.

Supervision as a component under the evidence for management and policy decision-making on MCHNP indicates that GHS is to provide the capacity to effectively coordinate, supervise, and monitor implementation of the community-based services. It is worth noting that the majority (80%) of the facilities had not received supervision on MCHNP from a higher level since the first quarter of 2019. By extension, this implies that the activities of the CHOs and volunteers are least supervised and supported regularly by senior managers. Also, opportunities to address barriers or bottlenecks and to provide timely feedback might be lost. Jaskiewicz contended that Community Health Workers (CHWs) have special supervision needs, usually because of the short duration of their training and mostly because they practice alone and are required to reach out to families in the communities [8]. Aikins and colleagues argued that the success of CHPS implementation depends largely on the effectiveness and frequency of facilitative supervision paid to CHOs by District and Subdistrict Health Management Teams [9]. It is therefore necessary to invest in high-quality supervision for CHWs to help them reach their full potential and help communities achieve optimum health.

Secondly, all facilities showed evidence of community registration of pregnant women, referrals of pregnant women to the next care level, sessions of community pregnancy care, provision of family planning services, and ensuring the availability of family planning commodities as prescribed in the implementation guidelines. The findings were found to be consistent with the 2011 Ghana Reproductive Health Strategic Plan [10], which emphasizes on evidence-based interventions that are most effective and can make a difference in the immediate and long-term well-being of women and newborns. These include improving facilities for women's access to antenatal, postnatal care, and family planning services so that maternal and newborn health status can be monitored, and timely interventions implemented, as necessary.

This study further demonstrated that implementers of the project executed fully all interventions under the child health improvement. There was evidence of bed net distribution and promotion of its usage to pregnant women and children below five years, counselling, and education and management of childhood illnesses at all CHPS centres studied.

Again, all facilities evaluated showed evidence of fully implementing nutrition interventions such as community-based education, infant and young child feeding and counselling, community-based growth promotion, home visits, and outreach services. The finding was found to be in line with a study by Majamanda et al. [11] on the effectiveness of community-based nutrition interventions, which identified growth monitoring and promotion, supplementary feeding, and education and communication for behaviour change as requirements for successful community-based nutrition intervention.

Barriers to smooth implementation of every project impact negatively on the realization of the project's objectives as planned. In this study, several barriers were identified from the perspective of the project implementers as mitigating against the smooth implementation, the majority of which were related to governance. All the participants identified the inadequate and delay in the release of funds as a major barrier that prevents them from embarking on their quarterly activities on schedule. It is also worth noting that all the participants complained of restrictions that come with the project fund which does not provide any minimal space for its usage on other mutually important activities but only for the purposes for which the funds were released, whether or not that particular activity was of higher priority at the time of the release. The importance of the adequate and timely release of funds for service delivery cannot be overemphasized. The World Health Organization, for instance, identifies three interrelated areas that are essential for achieving universal health coverage, which include providing enough fund for health care, reducing financial barriers to access to health, and allocating funds in a manner that promotes quality, efficiency, and equity [12]. Ensuring that these three domains exist will be crucial factors in determining the availability of essential health services to those who need them, irrespective of ability to pay.

Additionally, nearly 70% of the facilities evaluated did not have the MCHNP implementation guideline, which is the main strategic document for the implementation of the project, hence were not available for use. This suggests that implementers were not guided in their attempt to execute the project interventions to the beneficiaries. This could contribute to the poor performance of governance processes in the study since they might not be aware of what to put in place or do to improve governance indicators of the project.

Inadequate technical staff to carry out planned activities was also mentioned as a barrier, which results in extra workload for the few. This claim is corroborated by the background characteristics of the facilities studied, which showed an average of two CHOs per CHPS centres who are expected to carry out all the activities outlined in the implementation guidelines in addition to other routine activities. This situation stifles efforts to achieve better health outcomes. Workforce issues related to shortages and effective deployment of existing professionals need to be addressed before quality of care is further compromised. A major weakness in sub-Saharan African health systems is inadequate human resources. Africa is said to have less than one health worker per 1000 population compared to 10 per 1000 in Europe [13]. Health problems are worsened by shortages and unequal distribution of health professionals between urban and rural settings. In a study conducted by Tana [13], participants affirmed the insufficiency and inadequacy of health workers, which they described as leading to physical and mental exhaustion and in some cases to further deterioration of their medical condition. It is therefore critical to prudently manage and diversify health workforce particularly at the peripherals in order to ensure that community-led projects are well implemented.

Furthermore, regional and district MCHNP coordinators stated explicitly that there was no structured training or orientation for implementers before the project was rolled out. This shortfall was reemphasized by a finding in this study when only 10% of coordinators were able to enumerate correctly the objectives of the project. This will likely have an adverse influence on the project implementation as staff may not fully understand what to do and when. The need to increase the effectiveness and efficiency of health professionals through training and retraining cannot be overlooked. Lack of training may affect the quality of service delivery, hence should be taken as essential and prioritise for before the implementation of any new health programme. The rest of the barriers identified were poor community participation and apathy on the part of the District Health Directorate to support CHPS-based activities when MCHNP funds were depleted. Similarly, Belizan and colleagues in their study to identify the barriers and facilitators for the “Implementation and Evaluation of Community-Based Interventions” found that adequate funding, skilled personnel, equipment, and material resources; technical support for data management and analysis; training on project designs; political support from local; and acceptance of the proposed intervention by the local community were the main barriers to the project [14]. Haver et al. [15] also noted that staff inadequacy and mix, poor community engagements, and lack of motivation mitigate against smooth maternal and newborn service delivery.

Finally, this study observed a seemingly link between MCHNP governance scores and number of barriers mentioned by MCHNP coordinators and CHOs. This relationship may or may not represent causation between the two variables, but it does describe an existing pattern showing that the number of barriers increased whenever governance scores decreased. This finding shares a similar observation with Kickbusch et al. [16] in a study conducted for WHO on “Governance for health in the 21st Century” which indicated that “actors and activities of governance influence health programmes, however, global health actors today are largely unequipped to ensure that health concerns are adequately taken into accounts”.

The authors acknowledge the absence of vigorous statistical analysis to establish the true relationship between scores on the MCHNP processes and barriers enumerated as a limitation. This can be considered as a springboard for further studies. Potential limitation of this study may also include the inability to generalize findings to represent the entire country since only limited facilities in one region were involved in the study. Additionally, assessing the facilitators of the project implementation would have made this evaluation more complete, hence recommended to be considered in ensuing evaluation studies.

## 5. Conclusion

The evidence gathered by this study showed that maternal, child health, and nutrition activities by CHPS centres were implemented in accordance with the MCHNP implementation guidelines and shows very good implementation status for MCHNP service delivery. However, the status of governance is generally rated as partially completed. The main implementation barriers were related to funding and inadequate staff. We, therefore, recommend that

1. Governance processes in the MCHNP guideline should be reinforced across district and health facilities by the District and Regional Health Directorate
2. Review meeting should be organized by the Regional Health Directorate to engage District Directors, MCHNP coordinators, and Community Health Officers to identify and find solutions to barriers to the implementation of the project
3. Efforts should be made to maintain the current implementation status of maternal, child health, and nutrition processes of the project

## Figures and Tables

**Table 1:** Background characteristics of districts and CHPS facilities.

District	Expected pregnancy and children under 5	MMR/1000 LB	IMR/1000 LB	CHPS facilities evaluated	CHPS population	No. of years in operation	No. of CHOs	Average OPD attendants per month
Suhum	4,322	2	1	Akote	3,528	10	2	150
				Suhum Urban	5,675	10	3	283
Fanteakwa	5,104	3	2	Ehiamankyen	3,821	10	1	172
				Maine	2,978	8	1	78
New Juaben	2,968	5	2	Jumapo-Yeene	4,094	10	2	331
				Nyamekrom	3,786	3	2	91
Kwahu West	4,473	2	2	Wisiwisi	3,486	5	1	52
				Akramang	2,801	9	1	68
Nsawam	4,390	1	3	Chinto	3,137	6	2	50
				Dobro	3,275	8	2	83

MMR: maternal mortality rate; IMR: infant mortality rate; LBs: live births. Source: Eastern Regional Health Directorate 2018 Annual Report, Ghana Health Services.

**Table 2:** MCHNP governance processes scores by CHPS centres.

CHPS centres	MCHNP governance process indicators								Total score (out of 8)	% score	Status
	District focal persons	Prescribed cadre of focal persons	Able to identify objectives	Plans and budget	Quarterly meetings	Funds release mode	Funds accounting mode	Received supervision			
Akote	+	-	+	-	+	+	+	-	5	62.5	Partially completed
Suhum urban	+	-	-	-	-	+	+	+	4	50	Partially completed
Ehiamakyen	+	-	-	-	-	-	+	-	2	25	Incomplete
Maine	+	-	-	-	-	-	+	-	2	25	Incomplete
Jumapo	+	-	-	-	+	-	+	+	4	50	Incomplete
Nyamekrom	+	-	-	-	-	-	+	-	2	25	Incomplete
Wisiwisi	+	-	-	-	-	-	+	-	2	25	Incomplete
Akramang	+	-	-	-	-	-	+	-	2	25	Incomplete
Chinto	+	-	-	-	+	-	+	-	3	37.5	Incomplete
Dobro	+	-	-	-	-	-	+	-	2	25	Incomplete

Key: + = evidence available; - = no evidence.

**Table 3:** MCHNP maternal health processes scores by CHPS facilities.

CHPS centres	MCHNP maternal health process indicators						Total score (out of 6)	% score	Status
	Registration of pregnant women	Community referral	Antenatal care	Family planning for adolescents	Adolescent counseling	Family planning commodities in stock			
Akote	+	+	+	+	+	+	6	100	Fully completed
Suhum urban	+	+	+	+	+	+	6	100	Fully completed
Ehiamakyen	+	+	+	+	+	+	6	100	Fully completed
Maine	+	+	+	+	+	+	6	100	Fully completed
Jumapo	+	+	+	+	+	+	6	100	Fully completed
Nyamekrom	+	+	+	+	+	+	6	100	Fully completed
Wisiwisi	+	+	+	+	+	+	6	100	Fully completed
Akramang	+	+	+	+	+	+	6	100	Fully completed
Chinto	+	+	+	+	+	+	6	100	Fully completed
Dobro	+	+	+	+	+	+	6	100	Fully completed

Key: + = evidence available; - = no evidence.

**Table 4:** MCHNP child health processes scores at CHPS centres.

CHPS centres	MCHNP child health process indicators				Total score (out of 4)	% score	Status of child health process
	Distribution of bed nets	Health promotion on bed nets	Counseling on childhood illnesses	Management of childhood illnesses			
Akote	+	+	+	+	4	100	Fully completed
Suhum Urban	+	+	+	+	4	100	Fully completed
Ehiamankyen	+	+	+	+	4	100	Fully completed
Maine	+	+	+	+	4	100	Fully completed
Jumapo	+	+	+	+	4	100	Fully completed
Nyamekrom	+	+	+	+	4	100	Fully completed
Wisiwisi	+	+	+	+	4	100	Fully completed
Akramang	+	+	+	+	4	100	Fully completed
Chinto	+	+	+	+	4	100	Fully completed
Dobro	+	+	+	+	4	100	Fully completed

Key: + = evidence available; - = no evidence.

**Table 5:** MCHNP nutrition processes scores by CHPS facilities.

CHPS centres	MCHNP nutrition processes				Total score	% score	Status
	Community-based nutrition education	Counseling and promotion of IYCF	Community-based growth promotion sessions	Home visits and outreach services			
Akote	+	+	+	+	4	100	Fully completed
Suhum Urban	+	+	+	+	4	100	Fully completed
Ehiamankyen	+	+	+	+	4	100	Fully completed
Maine	+	+	+	+	4	100	Fully completed
Jumapo	+	+	+	+	4	100	Fully completed
Nyamekrom	+	+	+	+	4	100	Fully completed
Wisiwisi	+	+	+	+	4	100	Fully completed
Akramang	+	+	+	+	4	100	Fully completed
Chinto	+	+	+	+	4	100	Fully completed
Dobro	+	+	+	+	4	100	Fully completed

IYCF: infant and young child feeding. Key: + = evidence available; - = no evidence.

## Data Availability

The datasets used during the current study are available from the corresponding author on reasonable request.

## Abbreviations

BP: Blood pressure
CHN: Community Health Nurse
CHO: Community Health Officer
CHV: Community Health Volunteer
CHSP: Community-based Health Planning and Services
CHW: Community Health Worker
CMAM: Community-based management of acute malnutrition
DDHS: District Director of Health Services
DHS: Demographic and Health Survey
DHMT: District Health Management Team
ECNHA: Essential community nutrition and health actions
GHS: Ghana Health Services
IYCE: Infant and young child feeding
LMIC: Low- and middle-income country
MDG: Millennium Development Goal
RDHS: Regional Director of Health Services
SDG: Sustainable Development Goals
TFR: Total fertility rate
UN: United Nation.

## Supplementary Materials

Supplementary Materials SI 1 Supplementary material: Measurement indicators.
