# Supplementary material for: Process Evaluation of Maternal, Child Health and Nutrition Improvement Project (MCHNP) in the Eastern Region of Ghana: A Case Study of Selected Districts
Source: Biomed Res Int. 2020 Sep 18;2020:1259323. doi: 10.1155/2020/1259323 (PMC7520675; doi:10.1155/2020/1259323)
Supplement: Supplementary Materials — SI 1 Supplementary material: Measurement indicators. [file 1259323.f1.docx]

Measurement of indicators

| **Indicator** | **Measurement** | |
| --- | --- | --- |
| **Governance Processes**   1. Availability of MCHNP focal person. | Available / Not available | **Norm**  1 MCHNP focal person designated |
| 1. Category of staff identified as MCHNP focal person | Prescribed category / unprescribed category | District Directors of Health Services |
| 1. Knowledge on project Objectives. | Ability for MCHNP coordinators to identify MCHNP objectives. | Clarify all 3 objectives |
| 1. Availability of MCHNP plans and budget. | Available / Not available | Hard copy MCHNP specific action plan and budget for 1^st^ quarter 2019 available. |
| 1. Meetings held on MCHNP per quarter | Meeting records available/ Not available | At least 1 meeting in 1^st^ quarter 2019 held on MCHNP. |
| 1. Mode of release of MCHNP funds. | Approved mode or Non-approved mode | Cheque to district and CHPS Bank accounts |
| 1. Mode of accounting of MCHNP funds. | Approved mode or Non-approved mode | Funds held in trust ledger and  Advance ledger books. |
| 1. Monitoring and supervision received. | Evidence available / not available | At least 1 monitoring and supervision on MCHNP activities in a quarter. |
| **Maternal Health process**   1. Pregnant women registered in the first trimester. | Evidence available / not available | Availability of Register for the registration of pregnant women. |
| 1. Maternal health referrals made from the facility. | Evidence available / not available | Availability of records on maternal health referrals |
| 1. Community pregnancy care sessions held in the quarter. | Evidence available / not available | Availability of records on community pregnancy care sessions. |
| 1. Adolescents counseled on family planning. | Evidence available / not available | Availability of records on adolescents counselled on family Planning |
| 1. Family planning acceptors. | Evidence available / not available | Availability of records on family planning clients accepting a family planning method |
| 1. Availability of family planning commodities. | Available / Not available | Stock of family planning commodities. |
| **Child Health Processes**   1. Pregnant women and under-fives receiving bed nets. | Evidence available / not available | Availability of records on bed nets distribution to pregnant women and under-fives. |
| 1. Health promotion sessions on bed net use. | Evidence available / not available | Availability of records on promotion sessions on bed net |
| 1. Community-based counseling and education on childhood illnesses held. | Evidence available / not available | Availability of records on counselling and education on childhood illnesses. |
| 1. Under-fives managed for illnesses | Evidence available / not available | Availability of records on under-fives managed for illnesses. |
| **Nutrition Processes**   1. Community-based nutrition education held. | Evidence available / not available | Availability of records on community-based nutrition education sessions. |
| 1. Young Child and Infant (YCIF) feeding counseling sessions. | Evidence available / not available. | Availability of records on Young Child and Infant feeding counselling sessions |
| 1. Community based growth promotion sessions held. | Evidence available / not available | Availability of records on community-based growth promotion sessions. |
| 1. Outreach visits made in the quarter | Evidence available / not available | Availability of records on outreach visits per quarter. |
| 1. Barriers of MCHNP | Number various barriers enumerated by respondents | Not Applicable |
| 1. MCHNP implementation Status | Poor / Good/ Very good | Not Applicable |
